# Holy and Helping: The Role of Sanctification in the Communal Coping of Older African American Couples

**DOI:** 10.1093/geroni/igab046.1790

**Published:** 2021-12-17

**Authors:** Antonius Skipper, Andrew Rose, Jhazzmyn Joiner, Ethan Jones, Alex Reeves

**Affiliations:** 1 Georgia State University, Atlanta, Georgia, United States; 2 Texas Tech University, Lubbock, Texas, United States

## Abstract

Disproportionately affected by numerous relational stressors (e.g., financial strain, morbidity), older African American couples frequently find solace in religion and each other. Research notes that both married and cohabiting couples effectively respond to difficult situations by sharing the ownership of a stressor and organizing a collaborative, collective response. However, little is known about the influence of religion on shared coping experiences, particularly among older African American couples. This study examined dyadic data from the Strong African American Couples Project to capture the influence of relational sanctification on the communal coping practices of married and cohabiting older African American couples. The sample included 194 African American couples (146 married and 48 cohabiting) between the ages of 50 and 86 years. With the use of Actor Partner Independence Models, this study found that men’s sanctification predicted both their own communal coping and their partner’s communal coping. However, there were no significant effects when women’s sanctification was used as a predictor of communal coping among older African American couples. These findings are both important and novel, because these relationships had never before been examined within the United States, much less among older African American couples. Similar to existing research among majority White couples, this research finds that men’s religiosity may be a more influential predictor of relational outcomes than women’s religiosity. Such findings offer a valuable foundation for future studies seeking to consider how relational sanctification and communal coping may impact other outcomes associated with the romantic relationships of older African Americans.

